# Ocular Misalignment and Motility Limitation in Orbital Tumors

**DOI:** 10.1155/joph/4774755

**Published:** 2026-07-21

**Authors:** Min Seok Kang, Namju Kim, Hee Kyung Yang, Jeong-Min Hwang

**Affiliations:** ^1^ Department of Ophthalmology, Kyung Hee University College of Medicine, Kyung Hee University Hospital, Seoul, South Korea, khu.ac.kr; ^2^ Department of Ophthalmology, Seoul National University College of Medicine, Seoul National University Bundang Hospital, Seongnam, South Korea, snuh.org; ^3^ Department of Ophthalmology, Strabismus & Pediatric Ophthalmology Center, Kim’s Eye Hospital, Seoul, South Korea

**Keywords:** ocular misalignment, ocular motility, orbital tumor, strabismus

## Abstract

**Purpose:**

To predict prognosis and aid in the treatment of residual strabismus/ocular misalignment by analyzing the pattern of ocular motility disturbances caused by orbital tumors, and investigating factors associated with the severity of misalignment or motility limitation.

**Methods:**

The medical records of 63 patients diagnosed with orbital tumors were retrospectively reviewed. The location, type, and size of the tumor were noted. The limitation of extraocular muscle (EOM) movement was quantitatively scored for upgaze, downgaze, abduction, and adduction by the severity of limitation on a grading scale of 0 to –4. The maximum range of abduction, adduction, supraduction, and infraduction was measured by image analysis based on 9 gaze photographs using the 3D Strabismus Photo Analyzer.

**Results:**

Twenty patients (29%) experienced ocular motility limitations in the affected eye. Twelve patients had orbital tumors situated in the extraconal space (60%), while the other 8 patients (40%) had lesions in the intraconal space. The mean size of extraconal tumors was significantly larger than intraconal tumors (*p* = 0.010), and there were no significant differences in tumor size between every four groups. In 75% of patients with extraconal tumors, ocular motility was limited toward the action of the adjacent EOM. Conversely, in cases with intraconal tumors, ocular motility was limited toward the opposite direction of the nearest EOM action in 75% cases. Compared with the normal fellow eye, significant limitations of ocular motility were observed in adduction (*p* = 0.043) and supraduction (*p* = 0.002). The size of intraconal tumors was the only significant factor related to ocular motility limitation (*p* = 0.010).

**Conclusion:**

Ocular motility limitations were more common with large extraconal tumors, mostly in the direction of the adjacent EOM. In contrast, ocular motility was limited toward the opposite direction of the nearest EOM with intraconal tumors. Tumor size in the intraconal space was the only significant factor associated with ocular motility impairment.

## 1. Background

Orbital tumors can lead to various neurological and visual symptoms. They are classified based on their location and histological type. The anatomic location and the size of the tumor affect the initial signs and symptoms, sequelae, and available treatment options. In particular, various clinical symptoms may arise depending on adjacent intraocular structures surrounding the tumor. Cranial nerves and extraocular muscles (EOM) within the orbit can be affected by the tumor. As a result, patients with orbital tumors may experience discomfort such as diplopia caused by abnormal eye movement or misalignment, vision loss, and reduced sensory function [1–4]. The treatment approach should also take into consideration the anatomical location of the tumor and adjacent structures and nerves [5].

Orbital tumors vary in their occurrence by age group, and the rate of progression differs depending on whether they are benign or malignant. Sometimes, orbital tumors are easily overlooked in cases when there are no initial symptoms, and even when they do not progress rapidly. In some cases, ocular misalignment without notable pain or inflammation frequently serves as a key indicator for diagnosing an orbital tumor. The majority of orbital tumors typically result in ocular/globe malposition before or after attaining enough size to compress adjacent nerves [6–9]. Therefore, sudden onset of strabismus or ocular misalignment, as well as ocular motility limitations in a specific direction, can serve as crucial clinical manifestations for diagnosing orbital tumors [10].

However, to the best of our knowledge, there have been few studies that have quantitatively or qualitatively analyzed the limitations of ocular motility and/or ocular misalignment that arise in patients with orbital tumors, depending on the location or type of the tumor [11–19]. In the present study, we investigated the pattern of limitation in ocular movement and ocular misalignment in patients with orbital tumors and the factors associated with orbital tumors that influence ocular movement disorders. Therefore, the purpose of this study is to anticipate the occurrence or severity of symptoms such as diplopia or ocular motility disturbances caused by orbital tumors by factor analysis, and to assist in the treatment of residual diplopia or ocular misalignment after tumor therapy.

## 2. Materials and Methods

A retrospective review was performed on 63 patients who visited the Department of Ophthalmology, Seoul National University Bundang Hospital between the years 2006 and 2019 and were confirmed to have an orbital tumor. Patients who were diagnosed with thyroid orbitopathy, IgG4‐related ophthalmic diseases (ROD), and idiopathic orbital inflammation/pseudotumor were excluded. Patients with cranial nerve III, IV, or VI palsy that were not caused by orbital tumors, Brown syndrome, Duane retraction syndrome, or other restrictive strabismus were also excluded. Also, patients who were diagnosed with orbital tumor but had no records of a prism cover test or 9 gaze photographs—making it impossible to determine the presence or absence of strabismus or ocular motility limitation—were excluded. Informed consent was obtained from all subjects, and all medical procedures followed the tenets of the Declaration of Helsinki. The study protocol was approved by the institutional review board of Seoul National University Bundang Hospital (B‐2002‐592‐105).

The medical records of patients, including age, gender, ocular symptoms and signs, location of the primary orbital tumor and metastasis, tumor histology, treatment, and visual outcome, were reviewed. Ophthalmologic examinations, including visual acuity, ocular alignment with prism and alternate cover tests at 6 cardinal gazes, ductions and versions, pupillary examination, slit lamp examination, and fundus examinations were noted. Prism and alternate cover test at 6 m and 33 cm, respectively, in the primary position were used to measure the angle of deviation. The limitation of EOM movement was quantitatively scored for upgaze, downgaze, abduction, and adduction by the severity of limitation on a grading scale of 0 to −4. A grade of 0 was noted for full excursion, −4 for 0% excursion just reaching midline, and −3 to −1 for 25% increments [20]. In addition, we sought to use the 3D strabismus photo analyzer—which demonstrated reliability and reproducibility in measuring the angle of deviation in our previous study [21]—to quantify the degree of ocular motility limitation more objectively by measuring the angles of ocular deviation in right/left/up/down gaze. The maximum deviation of abduction, adduction, supraduction, and infraduction was measured by image analysis based on 9 gaze photographs. Considering that tumor types vary and that the exact boundaries and shapes of tumors are difficult to delineate based on imaging alone, the tumor size was determined using a one‐dimensional method by measuring the maximum diameter on the axial or coronal view of magnetic resonance imaging (MRI). In our cohort, although there were differences in the number and sequence of imaging studies performed, all patients underwent at least one MRI examination. Therefore, tumor size was analyzed based on MRI imaging.

A computed tomography (CT) scan was conducted using detector‐row machines (Philips Medical Systems, Cleveland, OH) with an intravenous nonionic contrast material (2 mL/kg; iopromide, Ultravist 370; Bayer, Berlin, Germany). Axial and coronal images were reconstructed with a 2 mm thickness at 3‐mm intervals. MRI was conducted using a 1.5‐tesla system (Gyroscan Intera; Philips, Healthcare, Best, the Netherlands) or a 3‐tesla system (Achieva; Philips, Healthcare, Best, the Netherlands) with a SENSE (SENSitivity Encoding) head coil. T1‐ and T2‐weighted imaging with gadolinium enhancement were performed to evaluate the orbit, including EOM. Abnormalities in orbital contents, including the EOM, were reviewed.

Tumor biopsy was performed when surgical removal was performed, or when biopsy was required for diagnosis, and the location was accessible. Patients with cavernous hemangioma and venous malformation that could be diagnosed solely based on MRI findings, as well as patients with optic nerve sheath meningioma and optic nerve glioma who demonstrated characteristic MRI findings and for whom biopsy carried a high risk of complications such as blindness, were excluded from biopsy.

Factors that can affect ocular motility limitation were evaluated by univariate and multivariate analyses. A *p* value of < 0.05 was considered statistically significant, and all analyses were performed with SPSS for Windows Version 26.0 (SPSS Inc., Chicago, IL).

## 3. Results

### 3.1. Patient Characteristics

The mean age of patients was 56.2 ± 17.8 (range, 6–91 years). Among them, 34 patients (54.0%) were males and 29 (46.0%) were females. The mean follow‐up duration was 32.9 ± 35.3 months (range, 2 weeks–148 months).

Fifty‐nine patients (93.7%) had unilateral involvement, and four patients (6.3%) had bilateral. Proptosis was the most common ocular symptom in 33 patients (52.4%), followed by eyelid swelling (15.9%), headache/eye pain (7.9%), diplopia (6.3%), visual impairment (4.8%), and epiphora (1.6%), while 7 patients had no symptoms and were incidentally found on imaging studies (17.9%). Ocular motility limitation was found in 20 patients (31.7%). Other signs included optic disc swelling (4.8%) and erythema/conjunctival injection (3.2%).

Orbital lymphoma was the most common type of tumor in 24 patients (38.1%), followed by cavernous hemangioma (17.5%), venous malformation (11.1%), optic nerve sheath meningioma (6.3%), osteoma/osteosarcoma (6.3%), pleomorphic adenoma (6.3%), schwannoma (6.3%), neurofibroma (1.6%), optic nerve glioma (1.6%), intraosseous meningioma (1.6%), rhabdomyosarcoma (1.6%), and histiocytoid carcinoma (1.6%).

The location of the tumor was in the extraconal space in 40 patients (63.5%), while 23 patients (36.5%) had intraconal tumors.

Sixty‐one patients (96.8%) had a primary orbital tumor. Only two patients (3.2%) had orbital metastasis from a primary tumor in the esophagus and kidney/retroperitoneum.

Thirty‐nine patients (61.9%) underwent treatment with surgical intervention or radiation therapy, such as gamma knife radiosurgery. Among those who were treated, 7 patients (17.9%) underwent both treatments simultaneously.

The patients’ mean visual acuity was logMAR 0.14 ± 0.26 at the initial visit, and after treatment or observation, the visual acuity at the final visit was logMAR 0.13 ± 0.17. During the follow‐up period, a total of four patients showed a significant change in visual acuity of more than four lines; among them, three patients experienced decreased visual acuity despite treatment, while one patient showed improvement.

### 3.2. Analysis of Tumor Size and Ocular Motility

Eight patients (12.7%) complained of diplopia when looking straight forward. Among all patients, the prism and alternate cover test were performed in 37 patients who complained of diplopia or ocular movement limitations in any direction of gaze. Among these 37 patients, 26 patients (70.3%) showed orthotropia (normal eye alignment), four (10.8%) had horizontal strabismus (eye misalignment in the horizontal plane), two (5.4%) had vertical strabismus (eye misalignment in the vertical plane), and five (13.5%) had both horizontal and vertical strabismus simultaneously.

**FIGURE 1:**
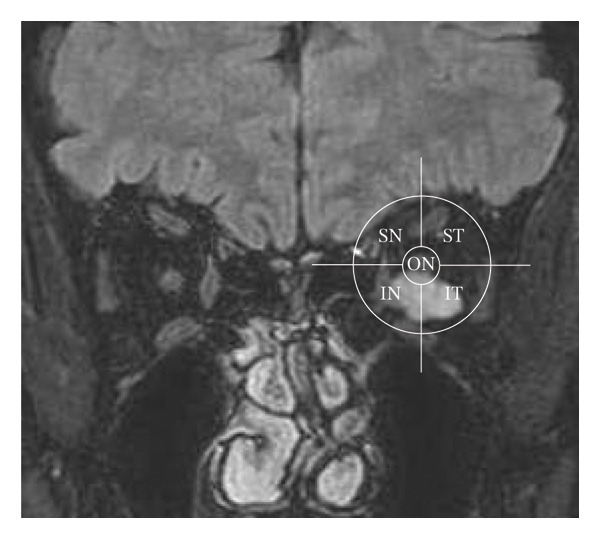
Analysis of tumor location divided into 4 segments determined by the mechanical axis of eye movement on MRI coronal imaging. ON = optic nerve, SN = superonasal, IN = inferonasal, IT = inferotemporal, ST = superotemporal.

The size of the tumor was measured and analyzed in 47 of 63 patients, excluding patients with multiple tumors or those for whom the measurement was difficult due to the ambiguous shape and boundaries of the tumor. The mean size of the tumor was 2.32 ± 1.29 cm (range, 0.5–8.0). According to the location of the tumor, the mean size was 2.79 ± 1.54 cm (0.9–3.5) in extraconal tumors and 1.84 ± 0.73 cm (0.5–8.0) in intraconal tumors. The size of extraconal tumors was significantly larger than intraconal tumors (*p* = 0.010) (independent *t*‐test). We analyzed the location of orbital tumors by dividing the orbit into four quadrants determined by the mechanical axis of eye movement on MRI coronal imaging: superonasal, inferonasal, inferotemporal, and superotemporal segments with the optic nerve as the central reference point (Figure 1). Orbital tumors confined to one quadrant were located superonasally in 12 patients, superotemporally in 12 patients, inferonasally in 5 patients, and inferotemporally in 2 patients. A total of 30 patients had orbital tumors involving two quadrants or more. Among them, 10 had tumors located in the superonasal and superotemporal quadrants, 7 in the superonasal and inferonasal quadrants, 6 in the inferonasal and inferotemporal quadrants, and 4 in the superotemporal and inferotemporal quadrants. Three patients had tumors extensively involving three or more quadrants. In addition, one patient with optic nerve sheath meningioma and one with glioma were not confined to specific quadrants.

To analyze the relationship between orbital tumors and ocular motility, we measured the distance between the tumor and each of the 4 extraocular rectus muscles, excluding oblique muscles. Based on these measurements, tumors closest to the superior rectus muscle were classified as “superior group,” those closest to the medial rectus muscle as “nasal group,” those closest to the inferior rectus muscle as “inferior group,” and those closest to the lateral rectus muscle as “temporal group.” Among all patients, 24 were classified in the superior group, 12 in the nasal group, 9 in the inferior group, and 4 in the temporal group. Two patients with optic nerve tumors, which were not associated with ocular motility limitation, and 12 patients with extensive tumors involving more than one rectus muscle—making ocular motility analysis difficult due to combined vector effects—were excluded from group classification. The mean size of tumor for each group was as follows: 2.76 ± 1.68 cm (0.5–8.0) in the superior group, 1.70 ± 0.73 cm (0.9–3.0) in the nasal group, 2.48 ± 0.99 cm (1.2–4.0) in the inferior group, and 2.35 ± 0.49 cm (2.0–2.7) in the temporal group. There were no significant differences in tumor size between each group (*p* = 0.200) (One‐way analysis of variance, ANOVA).

**FIGURE 2:**
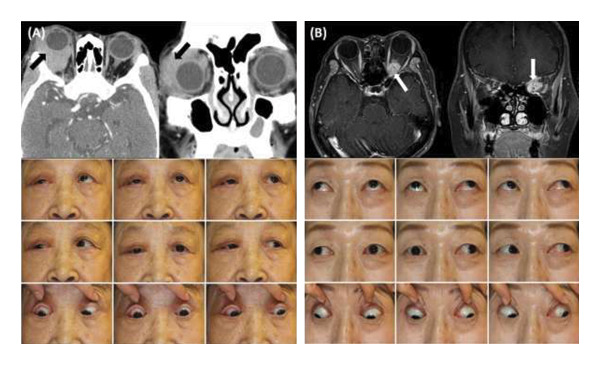
(A) Computed tomography (CT) showing lymphoma involving the right orbital cavity and lacrimal gland (black arrow). Nine gaze photos showing abduction limitation and ptosis in the right eye (case 1). (B) T1‐weighted magnetic resonance imaging (MRI) showing an optic nerve sheath meningioma in the left orbital apex (white arrow). Nine gaze photos showing adduction limitation in the left eye (case 19).

We evaluated ocular motility limitations based on the location of the tumor (Table 1, Figure 2). Twelve patients (60%) had orbital tumors located in the extraconal space, while the other 8 patients (40%) had tumors in the intraconal space. In cases of extraconal tumors, they were commonly situated in the superior and temporal positions, except for one case with lymphoma. On the other hand, intraconal tumors predominantly occupied the inferior and nasal positions. Contrasting outcomes were observed in terms of the tumor location classified into the four quadrants. Among the 12 patients with orbital tumors in the extraconal space, six patients (50%) had limitation on supraduction, two patients (16.7%) on adduction, one patient (8.3%) on abduction, and one patient (8.3%) on infraduction. Additionally, two patients (16.7%) who had ocular motility limitation in two or more directions also had extraconal tumors. Among the 8 patients with intraconal tumors, five patients (62.5%) had limitation on supraduction, one patient (12.5%) on abduction, one patient (12.5%) on adduction, and one patient (12.5%) on infraduction.

**TABLE 1:** Characteristics of 20 patients with ocular motility limitation.

Case	Sex	Age	Location		^∗^Quadrant	Group	Type	Treatment	EOM limitation	
									Direction	Grade
1	F	91	Extraconal	Superolateral orbit with unclear demarcation with the lacrimal gland and adhesion to the adjacent SR and LR	ST	Temporal	Lymphoma	CCRT	Abduction	−1
2	M	56		Lateral extraconal space	IT + ST	Temporal			Adduction	−2
3	M	68		Lateral extraconal space	IT + ST	Temporal			Adduction	−1
4	M	57		Between the orbital bony wall & SR	ST	Superior			Supraduction	−1
5	M	61		Between the orbital bony wall & SR, SR thickening	ST	Superior			Supraduction	−2
6	M	43		Between the orbital bony wall & SR, SR thickening	SN	Superior			Supraduction	−2
7	M	76		Between the orbital bony wall & SR	ST + SN	Superior			Supraduction	−1
8	M	37		Ill‐defined infiltrative lesion in the inferonasal orbit involving MR and IR with enhancement along the nasolacrimal duct	IN	Nasal			Multi	
9	M	30		Between the orbital bony wall and SR	SN	Superior	Neurofibroma	Surgery	Supraduction	−1
10	F	88		Superior orbital mass in the right frontal skull, destroying bone	SN + ST	Superior	Osteosarcoma	RT	Supraduction	−2
11	M	75		Lateral extraconal space	ST	Temporal	Pleomorphic adenoma	Surgery	Multi	
12	M	53		Enhancing ovoid mass outside of the superior orbit with dehiscence at the orbital roof	ST + SN	Superior	Schwannoma	Surgery	Infraduction	−2

13	M	62	Intraconal	Superomedial retrobulbar orbit	SN	Nasal	Lymphoma	—	Supraduction	−1
14	M	28		Between the optic nerve and the MR	SN + IN	Nasal	Cavernous hemangioma	Surgery	Abduction	−1
15	M	51		Between the optic nerve and IR/MR	IN	Inferior		—	Supraduction	−2
16	M	56		Between the optic nerve and IR	IN + IT	Inferior		GKRS	Supraduction	−4
17	M	42		Between the optic nerve and IR	IT	Inferior		—	Supraduction	−2
18	M	19		Between the optic nerve and SR	SN	Superior		—	Infraduction	−2
19	F	62		Orbital apex	ON	—	Optic nerve sheath meningioma	RT	Adduction	−1
20	M	34		Fusiform thickening of the intraorbital optic nerve	ON‐	—	Optic nerve glioma	RT	Supraduction	−3

Abbreviations: CCRT = concurrent chemoradiation therapy, EOM = extraocular muscle, GKRS = gamma‐knife radiosurgery, I = inferior, IN = inferonasal, IR = inferior rectus muscle, IT = inferotemporal, LR = lateral rectus muscle, MR = medial rectus muscle, N = nasal, RT = radiotherapy, S = superior, SN = superior nasal, SR = superior rectus muscle, ST = superior temporal, T = temporal.

^∗^When the tumor involved more than two quadrants, combined notation was used with a plus sign, and the quadrant with the greater extent was listed first.

The incidence of ocular dysmotility for each tumor location was as follows: 27% (8/29) in the superior group, 25% (3/12) in the nasal group, 33% (3/9) in the inferior group, and 100% (4/4) in the temporal group. Tumors in the temporal group significantly induced ocular dysmotility compared to other groups (*p* = 0.032) (Pearson’s chi‐square test).

We aimed to perform a more quantitative analysis of the relationship between orbital tumors and ocular motility limitation. In 20 patients with ocular motility limitation, we compared the affected eye with the contralateral normal eye. The maximum range of abduction, adduction, supraduction, and infraduction in both eyes were evaluated by image analysis of nine gaze photographs using an in‐house software that was developed in a previous study [13]. The affected eye exhibited a smaller range of motion compared to the normal eye in the direction of ocular limitation. In cases where there were limitations in two or more directions of eye movement, each direction was analyzed independently. Especially, in comparison between the normal fellow eye and affected eye, significant differences in ocular motility ranges were observed in supraduction (*p* = 0.002) and adduction (*p* = 0.043). However, no significant differences were found in abduction (*p* = 0.180) and infraduction (*p* = 0.068) (Wilcoxon signed rank test) (Table 2).

**TABLE 2:** Comparison of maximum ranges of ocular motility in the normal and affected eye.

EOM limitation	^∗^No. of patients (%)	Maximum range of ocular movement (degrees (°))		*p* value
		Normal eyes	Affected eyes	
Abduction	2 (8.7%)	37.8 ± 4.9	31.8 ± 5.3	0.180
Adduction	5 (21.7%)	37.9 ± 5.9	24.9 ± 8.9	**0.043**
Supraduction	12 (52.2%)	15.9 ± 6.2	9.8 ± 5.7	**0.002**
Infraduction	4 (17.4%)	46.4 ± 4.3°	34.3 ± 4.1	0.068

*Note:* EOM = extraocular muscle. Bold values indicate statistical significance (*p* < 0.05).

^∗^In 2 cases with restrictions in two or more directions of eye movement, each direction was analyzed independently.

Factors associated with ocular motility limitation were determined by univariate comparison (Table 3). In case of intraconal tumors, the size of the tumor in the group with motility limitations was larger than that in the group without limitations (*p* = 0.021) (independent *t*‐test). Regarding extraconal tumors, when the tumor was in the superior/temporal groups, the probability of ocular movement disorders was significantly higher compared to the inferior/nasal groups (*p* = 0.048) (Fisher’s exact test). By multivariate analysis, only the size of an intraconal tumor (*p* = 0.010) was a significant factor capable of causing ocular motility limitation (Table 4).

**TABLE 3:** Comparison of factors between groups with and without ocular motility limitation.

Factors			Motility limitation (+)	Motility limitation (−)	*p* value
Tumor size (cm)	All		2.74 ± 0.89	2.16 ± 1.40	^∗^0.174
	Extraconal		3.44 ± 0.59	2.62 ± 1.68	^∗^0.298
	Intraconal		2.30 ± 0.76	1.59 ± 0.59	^∗^ **0.021**

Tumor location (number)	All	S/T	12 (8 + 4)	21 (21 + 0)	^†^0.554
		I/N	6 (3 + 3)	15 (6 + 9)	
	Extraconal	S/T	11 (7 + 4)	11 (11 + 0)	^†^ **0.048**
		I/N	1 (0 + 1)	9 (5 + 4)	
	Intraconal	S/T	1 (1 + 0)	10 (10 + 0)	^†^0.056
		I/N	5 (3 + 2)	6 (1 + 5)	

*Note:* S = superior, N = nasal, I = inferior, T = temporal. Bold values indicate statistical significance (*p* < 0.05).

^∗^ *p* value by independent *t*‐test.

^†^ *p* value by Pearson’s chi‐square test or Fisher’s exact test.

**TABLE 4:** Multivariate regression analysis for ocular motility limitation.

Outcome	Variable		*B*	95% CI	*p* value
Ocular motility limitation	Tumor size		0.271	[0.034, 0.302]	**0.015**
	Tumor location	Extra/intraconal	−0.186	[−0.496, 0.213]	0.587
		Group	0.060	[−0.123, 0.062]	0.102
	Tumor type		0.843	[−0.238, 0.134]	0.342
	Gender		−0.327	[−0.918, 0.263]	0.974

*Note:* B = coefficient. Bold values indicate statistical significance (*p* < 0.05).

Abbreviation: CI = confidence interval.

## 4. Discussion

In this study, 20 of 63 patients (31.7%) exhibited limitation of eye movements in one or more directions. Among the 37 patients who underwent a prism and alternate cover test, 11 patients (29.7%) showed ocular misalignment in primary gaze, and 8 patients (72.7%) complained of diplopia. The most common direction of ocular motility limitation was upward, followed by adduction, downward, and abduction, respectively. Using the 3D Strabismus Photo Analyzer, the maximum range of movement in the eyes with a tumor was analyzed and compared with the normal fellow eyes. The results revealed a significant difference in range, particularly in supraduction and adduction. Specifically, it was found that the larger the size of intraconal tumors, the higher the likelihood of inducing ocular motility limitation.

Although orbital tumors have rare incidences, they have a substantial impact on morbidity and mortality rates. They consist of benign and malignant lesions with a wide spectrum of entities and can appear as primary, secondary, or metastatic tumors in pediatric and adult groups. Orbital tumors can manifest with acute or chronic onset and exhibit varying rates of progression. They can result in vision loss, ocular misalignment, deformity, and, in severe cases, even death [1–3]. Therefore, whenever an orbital tumor is suspected, imaging is mandatory. It is most important to determine the location of an orbital tumor through imaging evaluation, such as CT or MRI, because the anatomic location of such tumors affects the presenting signs and symptoms, sequela, and treatment approaches to be considered.

Ocular motility abnormalities can manifest either independently or as a secondary feature of an underlying systemic condition. It is important to note that causes of impaired eye movement can be easily missed or misdiagnosed. While orbital tumors can indeed be one of the causes that can lead to such ocular motility disorders, they tend to be relatively overlooked compared to other conditions that can cause ocular motility disorders, such as cranial nerve disorder, brain tumors or congenital disorders, inflammatory disorders including Tolosa–Hunt syndrome or IgG4‐related disease, vascular abnormalities including cavernous carotid fistula (CCF), and thyroid eye disease [3, 22, 23].

Generally, the tumor displaces the eye in the opposite direction. For example, inferomedial displacement of the eye is found in patients with lacrimal gland tumors, and intraconal tumors typically result in axial proptosis. In this way, orbital tumors can lead to globe malposition or ocular misalignment, resulting in limitation of EOM movement. However, in this study, there were differences in the pattern of limited eye movement depending on the location of the tumor. In cases of extraconal tumors, there was largely EOM limitation toward the action of the adjacent EOM (75%, 9 of 12 patients). On the other hand, in cases of intraconal tumors, there was a limitation of ocular motility toward the opposite direction of the adjacent EOM (75%, 6 of 8 patients) (Table 1). Considering the causes for these results, in the case of extraconal tumors, which are mostly located in the superotemporal orbit, it can be speculated that ocular motility limitation is related to the surrounding lacrimal gland, which can lead to compression of the adjacent superior rectus or lateral rectus muscle when involved. In our study, the presence of an extraconal tumor in the superior/temporal region was a significant indicator of inducing ocular motility disturbances compared to when it was located in the inferior/nasal position (*p* = 0.021). Additionally, bony destruction or dehiscence in the orbit can result in incarceration or displacement of the EOM, leading to subsequent movement limitation. We might regard this pattern of EOM limitation as the “paralytic pattern.” On the contrary, in the case of intraconal tumors, it can be hypothesized that cavernous hemangioma or glioma, which were the main cases in this study, lead to a mass effect in the relatively narrow intraconal space. It causes compression or displacement of the adjacent EOM. In fact, the size of intraconal tumors remained as the only factor related to ocular movement limitation in multivariate analysis (*p* = 0.010). Consequently, these actions result in a structural form where the adjacent EOM appears to be grasped or pulled, leading to a restrictive pattern of ocular motility limitation in the opposite direction of the affected EOM. In this way, we could consider this pattern as the “restrictive pattern.” In particular, cavernous hemangiomas typically do not exhibit progressive or active courses, resulting in a relatively longer duration of observation without active treatment compared to patients with extraconal tumors. Therefore, chronic progression of ocular motility restriction toward the opposite direction may be found in intraconal tumors.

The maximum range of ocular movement analyzed in this study was generally smaller compared to previous studies [24, 25]. However, it should be noted that the previous studies relied on analysis using Photoshop, whereas this study utilized a 3D photo analyzer with proven reliability in analysis. Therefore, the difference in range can be attributed to the methodological differences between the two studies. Furthermore, this study aimed to not only assess the maximum range of ocular movement but also investigate the presence of ocular misalignment and eye movement disturbance by comparing the range of angles between the healthy fellow eye and the eye with a tumor. This study, by analyzing the pattern of ocular motility limitation and identifying factors contributing to actual ocular movement disturbance, is expected to provide insights into the direction of treatment for strabismus or ocular misalignment in the future. Thus, the clinical significance of this study is considered to be substantial.

Limitations of this study include the retrospective nature of the review, a small number of patients, and variable follow‐up durations. Even considering the total of 63 patients, the sample size was small, and only 47 patients had tumors for which size analysis was feasible, representing an even smaller subset. Additionally, the fact that patients were recruited from a single institution could also be considered a potential factor that may induce biases. However, it should be noted that this study was conducted with a relatively small number of patients because orbital tumors are rare disorders, and many cases may not even present with strabismus or diplopia. Therefore, multicenter studies in follow‐up research could enhance the clinical significance and relevance by reducing biases through a larger cohort study. In addition, the follow‐up periods for the patients were not consistent, and there were instances of follow‐up loss or insufficient evaluation of ocular movement and ocular misalignment after tumor treatment. It was difficult to assess the recovery status or the presence of sequelae in patients with orbital tumors after treatment. Finally, we did not perform the forced duction test (FDT) or force generation test (FGT) of the affected eye with motility limitation. So, it may also be considered a drawback that we were unable to confirm the presence of a paralytic or restrictive patterns.

Mass lesions of the orbit can present with a wide range of clinical symptoms and manifestations. Intraconal masses originate within the orbit itself and typically result in gradual expansion, leading to proptosis and compressive optic neuropathy. On the other hand, extraconal lesions tend to arise from structures surrounding the orbit, such as the paranasal sinuses and lacrimal glands [3]. Depending on the location and size of the tumor, clinical symptoms can vary, and even the same symptoms can manifest with different grades of severity. It is important to recognize that strabismus/ocular misalignment and limitations in ocular movement can also be symptoms caused by orbital tumors.

In conclusion, according to the findings of this study, ocular motility limitations were more common with large extraconal tumors, mostly in the direction of the adjacent EOM. In contrast, ocular motility was limited toward the opposite direction of the nearest EOM with intraconal tumors. Tumor size in the intraconal space was the only significant factor associated with ocular motility impairment.

**Nomenclature**
EOM: Extraocular muscle
IgG4‐ROD: IgG4‐related ophthalmic diseases
CT: Computed tomography
MRI: Magnetic resonance imaging
CCF: Cavernous carotid fistula
FDT: Forced duction test
FGT: Force generation test

## Funding

This research did not receive any specific grant from funding agencies in the public, commercial, or not‐for‐profit sectors.

## Disclosure

Some part of this study was presented as an e‐poster at the European Academy of Neurology in June 2025.

## Ethics Statement

Informed consent was obtained from all subjects, and all medical procedures followed the tenets of the Declaration of Helsinki. This present study was approved by the institutional review board (IRB) of Seoul National University Bundang Hospital. IRB number is B‐2002‐592‐105.

## Consent

Please see the Ethics Statement.

## Conflicts of Interest

The authors declare no conflicts of interest.

## Data Availability

The datasets analyzed during the current study are not publicly available because public disclosure is not covered by the study protocol approved by the ethics committee, but they are available from the corresponding author upon reasonable request.
